# Meropenem vs standard of care for treatment of neonatal late onset sepsis (NeoMero1): A randomised controlled trial

**DOI:** 10.1371/journal.pone.0229380

**Published:** 2020-03-04

**Authors:** Irja Lutsar, Corine Chazallon, Ursula Trafojer, Vincent Meiffredy de Cabre, Cinzia Auriti, Chiara Bertaina, Francesca Ippolita Calo Carducci, Fuat Emre Canpolat, Susanna Esposito, Isabelle Fournier, Maarja Hallik, Paul T. Heath, Mari-Liis Ilmoja, Elias Iosifidis, Jelena Kuznetsova, Laurence Meyer, Tuuli Metsvaht, George Mitsiakos, Zoi Dorothea Pana, Fabio Mosca, Lorenza Pugni, Emmanuel Roilides, Paolo Rossi, Kosmas Sarafidis, Laura Sanchez, Michael Sharland, Vytautas Usonis, Adilia Warris, Jean-Pierre Aboulker, Carlo Giaquinto, on behalf of NeoMero Consortium

**Affiliations:** 1 Institute of Translational Medicine, University of Tartu, Tartu, Estonia; 2 INSERM SC10-US19, Villejuif, France; 3 Women's and Children's Health Department, Neonatal Intensive Care Unit, Azienda Ospedaliera-University of Padua, Padua, Italy; 4 Department of Neonatology, Neonatal Intensive Care Unit, Bambino Gesù Children’s Hospital, IRCCS, Rome, Italy; 5 University Department of Paediatrics, Immunological and Infectious Disease Unit, Bambino Gesù Children’s Hospital, IRCCS, Rome, Italy; 6 Sağlık Bilimleri Üniversitesi, Zekai Tahir Burak Kadın Sağlığı Eğitim ve Araştırma Hastanesi, Neonatoloji Kliniği, Ankara, Turkey; 7 Pediatric Highly Intensive Care Unit, Fondazione IRCCS Ca’ Granda Ospedale Maggiore Policlinico, Università degli Studi di Milano, Milan, Italy; 8 Department of Intensive Care, Tallinn Children’s Hospital, Tallinn, Estonia; 9 Paediatric Infectious Disease Research Group, Institute for Infection and Immunity, St George's University of London, London, United Kingdom; 10 3^rd^ Department of Pediatrics, Faculty of Medicine, Infectious Diseases Unit, Aristotle University School of Health Sciences, Hippokration Hospital, Thessaloniki, Greece; 11 Tartu University Hospital, Clinic of Anaesthesiology and Intensive Care, Tartu, Estonia; 12 2^nd^ Department of Neonatology, Faculty of Medicine, Aristotle University School of Health Sciences, Papageorgiou Hospital, Thessaloniki, Greece; 13 Neonatal Intensive Care Unit, Fondazione IRCCS Ca’ Granda Ospedale Maggiore Policlinico, Università degli Studi di Milano, Milan, Italy; 14 1st Department of Neonatology, Faculty of Medicine, Aristotle University School of Health Sciences, Hippokration Hospital, Thessaloniki, Greece; 15 Hospital Universitario Infantil LA PAZ- H. Carlos III, Madrid, Spain; 16 Faculty of Medicine, Vilnius University, Vilnius, Lithuania; 17 MRC Centre for Medical Mycology, Institute of Medical Sciences, University of Aberdeen, Aberdeen, United Kingdom; 18 Department of Women's and Children's Health, University of Padova, Padova, Italy; Universitatsklinikum Jena, GERMANY

## Abstract

**Background:**

The early use of broad-spectrum antibiotics remains the cornerstone for the treatment of neonatal late onset sepsis (LOS). However, which antibiotics should be used is still debatable, as relevant studies were conducted more than 20 years ago, recruited in single centres or countries, evaluated antibiotics not in clinical use anymore and had variable inclusion/exclusion criteria and outcome measures. Moreover, antibiotic-resistant bacteria have become a major problem in many countries worldwide. We hypothesized that efficacy of meropenem as a broad-spectrum antibiotic is superior to standard of care regimens (SOC) in empiric treatment of LOS and aimed to compare meropenem to SOC in infants aged <90 days with LOS.

**Methods and findings:**

NeoMero-1 was a randomized, open-label, phase III superiority trial conducted in 18 neonatal units in 6 countries. Infants with post-menstrual age (PMA) of ≤44 weeks with positive blood culture and one, or those with negative culture and at least with two predefined clinical and laboratory signs suggestive of LOS, or those with PMA >44 weeks meeting the Goldstein criteria of sepsis, were randomized in a 1:1 ratio to receive meropenem or one of the two SOC regimens (ampicillin+gentamicin or cefotaxime+gentamicin) chosen by each site prior to the start of the study for 8–14 days. The primary outcome was treatment success (survival, no modification of allocated therapy, resolution/improvement of clinical and laboratory markers, no need of additional antibiotics and presumed/confirmed eradication of pathogens) at test-of-cure visit (TOC) in full analysis set. Stool samples were tested at baseline and Day 28 for meropenem-resistant Gram-negative organisms (CRGNO). The primary analysis was performed in all randomised patients and in patients with culture confirmed LOS. Proportions of participants with successful outcome were compared by using a logistic regression model adjusted for the stratification factors. From September 3, 2012 to November 30th 2014, total of 136 patients (instead of planned 275) in each arm were randomized; 140 (52%) were culture positive. Successful outcome at TOC was achieved in 44/136 (32%) in the meropenem arm vs. 31/135 (23%) in the SOC arm (p = 0.087). The respective numbers in patients with positive cultures were 17/63 (27%) vs. 10/77 (13%) (p = 0.022). The main reason of failure was modification of allocated therapy. Treatment emergent adverse events occurred in 72% and serious adverse events in 17% of patients, the Day 28 mortality was 6%. Cumulative acquisition of CRGNO by Day 28 occurred in 4% of patients in the meropenem and 12% in the SOC arm (p = 0.052).

**Conclusions:**

Within this study population, we found no evidence that meropenem was superior to SOC in terms of success at TOC, short term hearing disturbances, safety or mortality were similar in both treatment arms but the study was underpowered to detect the planned effect. Meropenem treatment did not select for colonization with CRGNOs. We suggest that meropenem as broad-spectrum antibiotic should be reserved for neonates who are more likely to have Gram-negative LOS, especially in NICUs where microorganisms producing extended spectrum- and AmpC type beta-lactamases are circulating.

## Introduction

Despite significant changes in neonatal care over the last several decades, late onset bacterial sepsis (LOS) is still one of the leading causes of neonatal morbidity and mortality in developing but also in highly developed countries [1–3]. Although LOS is predominantly caused by coagulase negative staphylococci (CoNS) (36–66% of cases), Gram-negative rods are responsible for about 26–36% of cases [3, 4].

The early use of broad-spectrum antibiotic regimens remains the cornerstone for the treatment of LOS. However, which antibiotic regimen should be used is still debatable, as relevant studies in developed countries were conducted more than 20 years ago, were single centre or single country, insufficiently powered, evaluated antibiotics not in clinical use anymore and had variable inclusion/exclusion criteria and outcome measures [5, 6]. As a result, most antibiotics are prescribed off-label in neonates [7, 8] and treatment guidelines are based on expert opinion rather than on evidence from randomised controlled trials (RCT) [9]. As an example of this, we showed that 49 different antibiotic regimens were used for the empiric treatment of LOS in 111 patients across Europe [10]. In addition, there is significant variation in antibiotic dosing, including meropenem, in neonatal intensive care units (NICUs) [11]. The issue is now further complicated by the rise of antibiotic resistance in NICUs worldwide [12] and the paucity of new antibiotics entering the market [13–15].

Meropenem is a low protein-bound (2%), broad-spectrum carbapenem with activity against a wide variety of Gram-positive and Gram-negative bacteria including anaerobes and extended spectrum and AmpC chromosomal β-lactamase producing *Enterobacteriaceae*. Meropenem has been used off-label in NICUs for more than a decade [16] because of concerns around high rates of extended spectrum beta-lactamase producing enterobacteria and is now the second most commonly used antibiotic [11, 17]. The advantage of meropenem is its wider antibacterial coverage and thus potential of using monotherapy instead of combination therapy. However, there is serious concern around selection of carbapenem-resistant Gram-negative organisms (CRGNO)[18].

The safety and effectiveness of meropenem was recently evaluated in a single arm study including 200 infants < 91 days with suspected or confirmed intra-abdominal infections. In this study, however, only 11% of patients received meropenem as monotherapy and only 15% (29/200) had positive blood cultures. The study demonstrated that meropenem was well tolerated and efficacious [19]. Meropenem was included in the European Medicines Agency priority list of off-patent drugs for which studies in neonates are requested (http://www.ema.europa.eu/docs/en_GB/document_library/Other/2013/05/WC500143379.pdf).

The general aim of the study was to compare the efficacy and safety of meropenem with a predefined standard of care (SOC) antibiotic regimen for the treatment of LOS in patients admitted to NICU. The distribution of LOS-causing microorganisms and their antibiotic susceptibility, relapse- and new infection rates, short-term outcome of LOS and mucosal colonisation with CRGNO were also evaluated.

## Methods

### Population and sample size calculations

NeoMero-1 was a randomised, open-label study conducted in 18 NICUs in Estonia, Greece, Italy, Lithuania, Spain and Turkey [20].

Patients eligible for the study had to meet following inclusion criteria: postnatal age (PNA) between 72 hours and ≤ 90 days and clinical or culture proven LOS. Culture confirmed LOS was defined as the presence of at least one positive culture from a normally sterile site together with at least one abnormal clinical or laboratory parameter within the 24 hours prior to randomisation as demonstrated in Table 1 [20]. Clinical sepsis criteria were based on postmenstrual age (PMA). If PMA was > 44 weeks the International Paediatric Sepsis Consensus Conference criteria had to be met [21]. For patients with PMA ≤44 weeks the criteria defined by the European Medicines Agency Expert Meeting on Neonatal and Paediatric Sepsis [5, 20] were used and the presence of at least two clinical and two laboratory parameters within the 24 hours prior to randomisation were required (Table 1).

**Table 1:** Clinical and laboratory parameters defining LOS in patients with PMA ≤ 44 weeks within the 24 hours prior to randomization.

**Clinical parameters**
1. hyper- or hypothermia or temperature instability; 2. reduced urinary output or hypotension or mottled skin or impaired peripheral perfusion; 3. apnea or increased oxygen requirement or need for ventilatory support; 4. bradycardia spells or tachycardia or rhythm instability; 5. feeding intolerance or abdominal distension; 6. lethargy or hypotonia or irritability; 7. skin and subcutaneous lesions (such as petechial rash or sclerema)
**Laboratory parameters**
1. white blood cell count < 4 or > 20 x 10^9^ cells/L; 2. immature to total neutrophil ratio > 0.2; 3. platelet count < 100 x 10^9^/L; 4. C-reactive protein > 15 mg/L or procalcitonin ≥ 2 ng/mL; 5. glucose intolerance when receiving normal glucose amounts (8–15 g/kg/day) as expressed by blood glucose values > 180 mg/dL or hypoglycemia (<40 mg/dL) confirmed on at least two occasions; 6. acidosis with base excess (BE) < -10 mmol/L or lactate above 2 mmol/L

Exclusion criteria included administration of any systemic antibiotics for more than 24 hours within the 7 days prior to randomisation unless the change was driven by lack of efficacy, LOS caused by microorganisms suspected or known to be resistant to study antibiotics, severe congenital malformations if the baby was not expected to survive for more than three months, presence of renal failure and/or requirement of hemofiltration or peritoneal dialysis and known intolerance of study medication.

#### Sample size calculation

On the limited published data available, we estimated that success rate in the control arm would be 64% [2]. The required sample size to show an absolute increase of success rate by at least 13% (from 64% to 77%) with 80% power in the meropenem arm using a 2-sided test at an alpha level of 0.05 was 220 patients per arm. Using a clinical definition of LOS, an ineligibility rate of 15% to 20% was anticipated. The sample size was thus conservatively increased to 275 subjects per arm to compensate for the dilution effect. The study had issues with recruitment and was closed on November 30th, 2014 with 272 patients randomised, due to expiration of funding by the European Commission. Given an unexpected overall low rate of success (30% instead of 70% due to frequent modifications of allocated therapy) and the very low percentage of subjects not having LOS (1.8%), the original planning assumptions seemed to be violated. Future trials should address such deviations during the planning process.

### Randomisation

Patients were centrally randomised using a computer generated randomisation list (1:1 ratio) and allocated to either meropenem or one of the two SOC regimens (ampicillin + gentamicin or cefotaxime + gentamicin) chosen by each site prior to the start of the study. The randomization list was prepared by the trial statistician before the beginning of the trial, with the SAS^®^ software (version 9.3), using computerized random numbers. Randomization was blocked and stratified by SOC regimen and use of systemic antibiotics for LOS within 24 hours prior to randomisation. The randomization list was kept confidential to trial team and sites received automated treatment assignment centrally via the e-CRF. No masking was used.

### Procedures

Meropenem was given via 30-minute intravenous infusion at a dose of 20 mg/kg q8h with the exception of those with gestational age (GA) < 32 weeks and PNA <2 weeks who received the same dose q12h with the possibility to increase dosing frequency to q8h from a PNA of two weeks. Ampicillin, cefotaxime and gentamicin were administered according to the British National Formulary for Children (BNFC, www.bnfc.org). Total duration of allocated therapy was predefined as 8 to 14 days. The concomitant use of other systemic antibiotics was not allowed with the exceptions of vancomycin, teicoplanin or linezolid, if started between screening and randomisation. The use of topical anti-infectives, systemic antifungals, antivirals, immunoglobulins and probiotics was permitted.

Patients were examined at Day 0 (screening and randomisation), Day 3, end of allocated therapy (EOAT), end of antibacterial therapy (EOT) and test of cure (TOC) visit, which was performed 2 ± 1 days after EOT for patients treated with antibiotics for the predefined duration (11 ± 3 days). Short-term follow-up visit was performed on Day 28 by on-site visit or telephone call for all surviving patients.

To avoid bias of the open label study, the components of the primary endpoint were assessed by the investigator as described below in outcomes section. Investigators assessed resolution or improvement of the clinical and/or laboratory parameters based on predefined algorithms. Investigators assessement was cross-checked againts data on clinical examination, laboratory parameters, microbiological assessments, etc. to verify correctness of all components of the primary endpoint.

Microbiological samples from normally sterile sites were taken at baseline, Day 3, on appearance of any new signs suggestive of LOS and repeated until the relevant microorganisms were no longer detected. All samples were processed at local laboratories according to their own laboratory procedures. In a post-hoc analysis two experts (IL and JG) reviewed the susceptibility data and categorised organisms as susceptible, non-susceptible to study antibiotics, or not possible to categorise. Rectal swabs were collected within 72 hours of baseline, at EOT and at Day 28 visit or NICU discharge, and stored locally at -80°C before being periodically transferred to the central Biobank. The samples were then sent in regular batches to St George’s, University of London, Department of Medical Microbiology. The thawed faecal samples were cultured using selective media and tested for carbapenem resistance according to EUCAST guidelines (http://www.eucast.org/ast_of_bacteria/guidance_documents). The isolate was considered CRGNO if phenotypic resistance was detected to meropenem or if *Stenotrophomonas maltophilia* was isolated, and to be highly CRGNO if meropenem MIC values were ≥8 mg/L. Acquisition of CRGNO during the study was defined if these microorganisms were not detected at baseline but were found in subsequent colonisation cultures.

Hearing was assessed according to local audiological protocols between EOT and Day 28 visit.

Cerebral ultrasound (and if persistently abnormal, magnetic resonance imaging or computed tomography) was undertaken at any time between EOT and Day 28 visit.

Blood and cerebrospinal fluid samples were collected for pharmacokinetic assessment; the results of this are reported separately [22].

### Outcomes

The composite primary endpoint was assessed at the TOC visit by the investigators and defined as success if (1) the patient was alive, and (2) all baseline clinical and laboratory parameters that defined LOS were resolved or significantly improved, and (3) the baseline microorganisms were eradicated or presumably eradicated with no new microorganisms identified, and (4) allocated therapy was given for 11 ± 3 days without any modification for more than 24 hours. All other situations were considered failures.

The secondary outcomes were safety, clinical and laboratory response on Day 3, at EOAT and EOT, survival at Day 28, time to NICU discharge, and presence of hearing disturbances and abnormalities in brain ultrasound, acquisition of CRGNO in rectal swabs and occurrence of relapses or new infections after successful outcome at TOC visit until Day 28. Resolution or improvement of clinical and laboratory parameters was evaluated by the study statistician by using predefined algorithms. Clinical relapses were defined as recurrence of LOS together with initiation of a new course of antibiotic treatment, and microbiological relapse as an isolation of a phenotypically similar organism from a normally sterile site in a patient with signs of infection. An adverse event (AE) was defined as any untoward medical occurrence or deviation of laboratory parameters of any causality in a patient receiving study treatment. Severity of the AEs was assed according to the Common Terminology Criteria for Adverse Events v3.0 (CTCAE); published 9.August 2006 (http://ctep.cancer.gov).

### Statistical analysis

The primary analysis included all randomised patients (full analysis set—FAS). Analysis of the primary endpoint was also performed in patients with culture confirmed LOS. Proportions of participants with successful outcome were compared by using a logistic regression model adjusted for the stratification factors. Additional efficacy analyses were performed by ignoring the changes in allocated therapy due to safety reasons or all changes of allocated therapy and by allowing duration of allocated therapy between 7 and 14 days. Other efficacy endpoints included clinical response at Day 3, end of allocated therapy and EOT, new infection and/or relapse by day 28. Safety analysis included all patients that received at least one dose of allocated therapy.

All analyses were performed with the use of SAS software, version 9.3 (SAS institute). Survival at day 28 was described using Kaplan-Meier method and curves were compared using a log rank test. A significance level of 5% was used and all p-values were the results of two sided tests. Binary variables were compared using chi-square test or an exact test as appropriate. For quantitative variables, Student’s t-test or Wilcoxon rank-sum test as needed were employed for comparisons between the treatment groups.

### Ethics and registration

The informed consent was signed by parents/guardians prior to randomisation. The following local ethics committees approved the study: Comitato Etico per la Sperimentazione c/o Servizio di Farmacologia Comitato Etico per la Sperimentazione della provincia di PADOVA, Comitato Etico per la Sperimentazione Clinica della provincia di TREVISO, Comitato Etico per la Sperimentazione del Farmaco Ospedale pediatrico Bambino Gesù, Comitato di Bioetica Dell'Ospedale S. Giovanni Calibita Fatebenefratelli—Isola Tiberina di ROMA, Comitato di Bioetica della Provincia Romana Comitato etico Interaziendale Novara, Comitato Etico della Provincia di MODENA, Comitato Etico di Bari, Comitato di Etica e Spermentazione Farmaci, Comitato Etico per la Sperimentazione Clinica della provincia di VICENZA, Comitato di Etica e Spermentazione Farmaci Comitato Etico Asl Roma C, Research Ethics Commitee of the University of Tartu, CEIC Hospital LA PAZ, The Lithuanian Bioethics Committee, National Ethics Committe for Greece, Zekai Tahir Burak Maternity Research and Training Hospital Clinical Research Ethics Committee.

The study was overseen by an independent data safety monitoring board and was registered in EudraCT database (2011-001515-31) and in clinicaltrials.gov (NCT01551394).

## Results

### Study population and baseline characteristics

**Fig 1:**
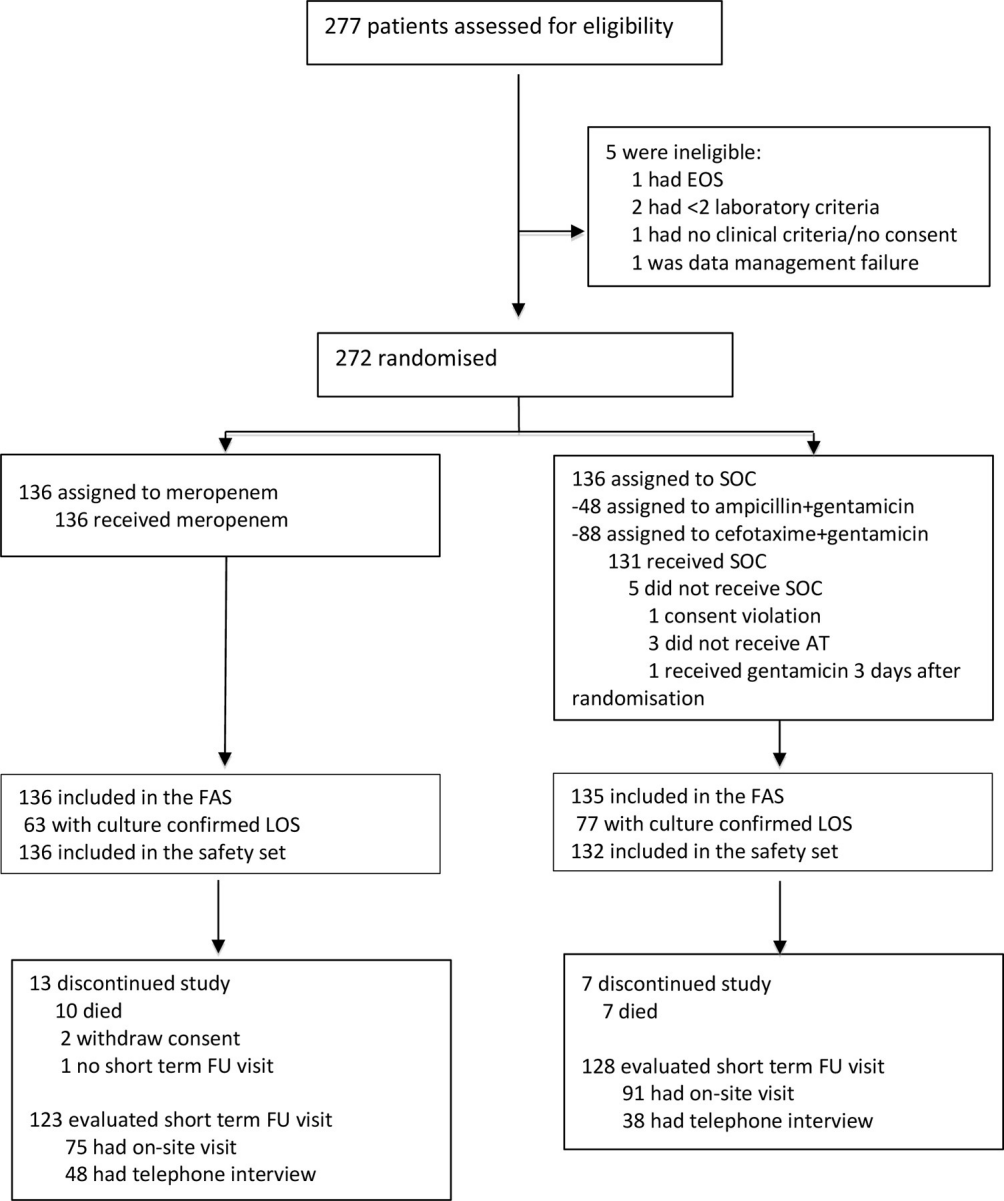
Flowchart of the study NeoMero-1. EOS–early onset sepsis; SOC–standard of care; FAS–full analysis set; AT–allocated therapy; LOS–late onset sepsis; FU–follow-up.

A total of 277 infants were assessed for the eligibility and 136 in each arm underwent randomization from September 3rd 2012 to November 30th 2014. In the SOC arm 48 (35%) patients were assigned to ampicillin + gentamicin and 88 (65%) to cefotaxime + gentamicin (Fig 1). One patient with a major informed consent violation in the SOC arm was excluded leaving 271 patients to be analysed for efficacy. There were 265 (98%; 132 in meropenem and 133 in SOC arm) with LOS and 140 (52%) of them had culture proven LOS. There were 268 (99%) patients who received at least one dose of allocated therapy and were included in the safety analysis.

The baseline characteristics of patients were well balanced between both arms (Table 2). They were also similar when patients were sub-grouped according to prior antibiotic treatment, culture proven LOS or presence of Gram-positive or Gram-negative LOS. Patients in the ampicillin+gentamicin sites were more gestationally mature than those in the cefotaxime+ gentamicin sites—median PMA 39.8 weeks (IQR 34.1; 42.5) vs. 32.3 weeks (IQR 29.4; 36.4) and median BW 2560g (IQR 1135; 3200) vs. 1105g (IQR 810; 1850), respectively.

**Table 2:** Characteristics of study population at baseline (FAS population). Data are presented as numbers (%) if not stated otherwise.

Characteristic	Meropenem N = 136 (%)	SOC N = 135 (%)
**Demographics**		
Median GA weeks (IQR)	31.6 (26.4–37.3)	30.6 (27.0–36.3)
<28 weeks	41 (30%)	41 (30%)
28–32 weeks	31 (23%)	38 (28%)
32–37 weeks	26 (19%)	23 (17%)
≥37 weeks	38 (28%)	33 (24%)
Median PNA days (IQR)	16 (8–30)	16 (8–30)
Median PMA days (IQR)	34.5 (30.5–40.7)	33.8 (29.9–40.1)
PMA > 44 weeks n (%)	5 (3.7%)	6 (4.4%)
Male n (%)	72 (53%)	72 (53%)
Median (IQR) birth weight (g)	1540 (840–2830)	1340 (850–2530)
-BW <1000 g (n)	45 (33%)	51 (38%)
-BW <1500 g (n)	67 (49%)	80 (59%)
-BW >2500 g (n)	43 (32%)	37 (27%)
SGA *n (%)	33 (24%)	34 (25%)
**Peri- or neonatal conditions**		
Multiple births	29 (21%)	32 (24%)
Medically assisted fertilisation	21 (16%)	15 (11%)
Antenatal steroids	65 (48%)	71 (53%)
Congenital conditions:		
-Respiratory	18 (13%)	17 (13%)
-Cardiovascular	13 (10%)	11 (8%)
-Gastrointestinal	8 (6%)	10 (7%)
-Neurological	8 (6%)	4 (3%)
-Other	6	6
Surgery	23 (17%)	29 (21%)
Arterial catheters	27 (20%)	32 (24%)
Central Venous Catheter	64 (47%)	69 (51%)
Mechanically ventilated	75 (56%)	74 (55%)
Received antibiotics prior to randomisation	100 (74%)	98 (73%)
Median duration of prior antibiotic therapy (hours)	18.5 (9.0–22.1)	16.0 (8.3–21.2)
Received meropenem prior to randomisation	35 (26%)	29 (21%)

* defined by birth weight ≤ 10^th^ percentile; IQR–interquartile range.

GA–gestational age; PNA–postnatal age; PMA–postmenstrual age; BW–birth weight; SGA–small for gestational age.

In total 200 (74%) patients were premature (35% with birth weight <1000 g) and only 11 had a PMA >44 weeks. In the 24 hours prior to randomisation 73% of patients had received antibiotics; 24% had received meropenem with a similar frequency in both study arms (Table 2).

**Fig 2:**
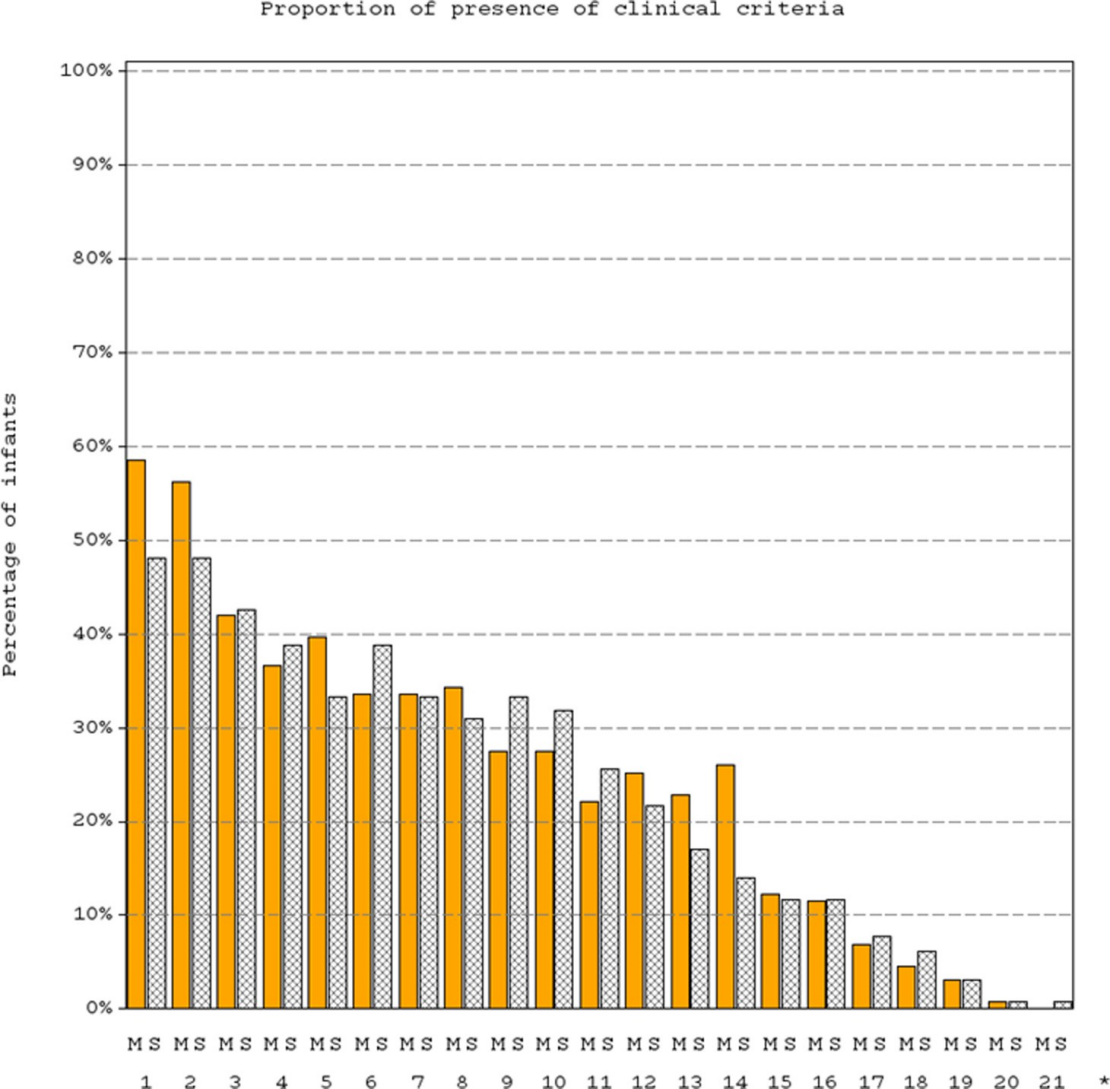
Distribution of clinical criteria of LOS at baseline in patients of PMA < 44 weeks in meropenem (M) and SOC (S) arm. The numbers represent the following clinical signs: **1-** Impaired peripheral perfusion, **2-** Mottled skin, **3-** Feeding intolerance, **4-**Apnoea, **5-**Increased oxygen requirement, **6-** Requirement for ventilation support, **7-** Abdominal distension, **8-** Hypotonia, **9-**Tachycardia, **10:** Lethargy, **11:** Bradycardia spells, **12:** Hyperthermia, **13:** Hypothermia, **14:** Hypotension, **15:** Other skin and subcutaneous lesions, **16:** Irritability, **17:** Rhythm instability, **18:** Reduced urinary output, **19:** T° instability, **20:** Petechial rash, **21:** Sclerema.

Patients of PMA ≤44 weeks had a median (IQR) of 3 (3–4) clinical and 2 (2–3) laboratory signs at baseline, in both arms. Clinical or laboratory signs that were seen in more than 50% of patients included impaired peripheral perfusion, mottled skin, CRP >15 mg/L and lactate >2 mmol/L (Fig 2).

### Aetiology of LOS

Baseline blood cultures were positive for 63/132 (46%) patients in the meropenem and 77/135 (57%) in the SOC arm with no differences in species distribution between study groups (Table 3).

**Table 3:** Causative agents of late onset sepsis and their susceptibility to study antibiotics.

Microorganism	Meropenem		SOC	
	Total N = 63 (%)	Susceptible to meropenem N (%)	Total N = 77 (%)	Susceptible to ≥1 antibiotic of SOC N (%)
**Gram-positive organisms**	**31 (49)**	**8 (26)**	**44 (57)**	**12 (27)**
CoNS	22 (35)	3 (14)	35 (45)	4 (11)
-*S*. *epidermidis*	14 (22)	2 (14)	25 (32)	4 (16)
-Other CoNS	8 (13)	1 (13)	10 (13%)	0
*S*. *aureus*	5 (8)	3 (60)	5 (6)	5 (100)
-MRSA	2 (3)	0	1 (1)	1 (100)
GBS	2 (3)	2 (100)	3 (4)	3 (100)
*Enterococcus*	1 (2)	0	1 (1)	0
Other Gram positives	1 (2)	0	0	-
**Gram-negative organisms**	**24 (38)**	**22 (92)**	**25 (32)**	**18 (72)**
*Enterobacteriaceae*	22 (35)	20 (91)	21 (27)	16 (76)
- -*Enterobacter* spp.	8 (13)	7 (78)	10 (13)	6 (55)
- -*K*. *pneumoniae*	7 (11)	6 (86)	4 (5)	3 (75)
- -*K*. *oxytoca*	4 (6)	4 (100)	3 (4)	3 (100)
- -*Serratia* spp.	0	-	1 (1)	1 (100)
Non-fermentative	2 (3)	2 (100)	2 (3)	1 (50)
- -*Pseudomonas* spp.	2 (3)	2 (100)	2 (3)	1 (50)
Other Gram-negative	0	-	2 (3)	1 (50)
**Mixed**	**8 (13)**	**2 (25)**	**8 (10)**	**2 (25)**

All differences non-significant between study arms; GBS–group B streptococci; CoNS–coagulase negative staphylococci; MRSA–methicillin resistant *S*. *aureus;* SOC–standard of care.

Of all Gram-negative microorganisms a total of 46 (94%) were susceptible to meropenem, 17 (59%) to cefotaxime, 2 (4%) to ampicillin and 32 (65%) to gentamicin. Altogether 32/63 (51%) of all microorganisms in the meropenem and 32/77 (42%) in the SOC arms were susceptible to the allocated antibiotics.

### Antibiotic treatment

Allocated therapy was used according to the protocol in 134 (99%) of patients in the meropenem and 127 (94%) in SOC arm. In total, 65 (48%) and 67 (50%), received allocated therapy alone and 69 (51%) and 58 (43%) received concomitant glycopeptides in the meropenem and SOC arms, respectively. The median duration of allocated therapy was comparable in both arms (7.9 [IQR 4.0–9.7] days in the meropenem vs. 7.0 [IQR 2.5–9.6] days in the SOC arm; p = 0.089) but the duration of any antibiotic therapy was shorter in the meropenem than in the SOC arm (9.0 [IQR 7.8–12.0] vs. 10.4 [IQR 8.5–13.3] days, respectively; p = 0.0085)

### Primary efficacy analysis

In the FAS population the primary outcome (i.e. the proportion of patients with a successful outcome at TOC) was comparable in both study arms—44/136 (32%) in meropenem vs. 31/135 (23%) in SOC arms (p = 0.087) (Table 4).

**Table 4:** Primary outcome–response at TOC visit. Data are presented as numbers (%).

	Meropenem n = 136 (%)	SOC N = 135 (%)	p-value
Successful outcome	44 (32)	31 (23)	0.087*
Alive	126 (93)	129 (96)	
Resolution or significant improvement of clinical findings and no new signs**	56 (41)	52 (39)	
Confirmed or presumed microbiological eradication**	71 (52)	73 (54)	
Completed allocated therapy without modification	58 (43)	46 (35)	

*logistic regression model including factors of stratification.

** assessed only if antibiotic therapy was given for 8–14 days (n = 74 in the meropenem and n = 76 in the SOC arm)

In culture-confirmed LOS the successful outcome at TOC visit was greater in meropenem as compared with SOC arm (17/63 (27%) vs. 10/77 (13%), respectively; p = 0.02, OR 95% CI: 3.0 (1.2–7.5).

Far the most common reason for failure was modification of allocated therapy, which was numerically more frequent in the SOC than in the meropenem arm—78/136 (57%) vs. 85/135 (63%), respectively. Other reasons for failure in meropenem and SOC arm, respectively were clinical signs not resolved or new signs occurring (13% vs. 18%), death prior to TOC (7% vs. 4%), microbiological failure (2% vs. 1%) and antibiotics not started or not allowed antibiotics given (1% vs. 7%).

In the meropenem arm modification of allocated therapy was mainly due to completion of allocated therapy before Day 8 (38%) and diagnosis of meningitis (13%), while isolation of resistant microorganisms (19%), lack of clinical response (18%) and use of inappropriate study antibiotics (18%) were the most common reasons in the SOC arm (Table 5).

**Table 5:** Reasons for modification or discontinuation of allocated therapy.

	Meropenem N = 78 (%)	SOC N = 85 (%)	Median duration of allocated therapy (days; IQR)
Treatment completed before Day 8	30 (38)	10 (12)	7.6 (7.0–7.7)
Meningitis diagnosed	10 (13)	7 (8)	1.1 (0.2–1.7)
Lack of response	8 (10)	15 (18)	3.1 (0.8–4.6)
Introduction of new and/or continuation of antibiotics after EOAT	8 (10)	5 (6)	9.7 (8.6–12.7)
*Study antibiotics not needed based on culture results	5 (6)	15 (18)	3.0 (2.4–4.4)
Death	4 (5)	3 (4)	1.5 (0.2–5.0)
Adverse event	4 (5)	4 (5)	1.9 (1.3–2.7)
Resistant microorganism isolated	3 (4)	16(19)	2.9 (2.2–4.9)
Treatment completed after Day 14	1 (1)	2 (2)	15.0 (14.8–16.4)
Other	5 (6)	8 (9)	4.1 (1.9–5.2)

*All but one patient had CoNS and 1 case had methicillin susceptible *S*. *aureus*.

In a posthoc analysis that allowed a duration of allocated therapy between 7 and 14 days (instead of 8 to 14 days), a successful outcome was more frequent in the meropenem than in the SOC arm (65/136, 48% vs 37/135, 27%; p = 0.001 in FAS and 26/63, 40% vs 15/77, 19%; p = 0.005 in culture confirmed sepsis population). There were no differences in success rate between meropenem and SOC arms if changes in the allocated therapy for safety reasons (success 44/136; 32% vs 31/135; 23%, respectively) or if all modifications of allocated therapy were ignored (success 54/136; 41% vs 49/135; 37%, respectively). This indicates once again that differences between study groups in response rates were mainly triggered by the modification of allocated therapy.

The success rate was greater for infants with Gram-negative than those with Gram-positive LOS (28% vs 13%; p = 0.046) mainly because of the modification of allocated therapy. The success rate in Gram positive sepsis was 21% in meropenem vs 7% in SOC arm and in Gram negative sepsis 34% vs 23%, respectively; these differences were not statistically significant. The influence of vancomycin as empiric therapy at baseline was tested in a log-binominal model but it did not significantly influence the primary outcome.

### Secondary analysis and short term outcome

In terms of secondary endpoints both study arms were similar as presented in Table 6.

**Table 6:** Secondary endpoints.

	Meropenem n/N (%)	SOC n/N (%)	P =
Success in patients infected with microorganisms susceptible to at least one component of allocated therapy	13/32 (41)	10/32 (31)	0.176
Clinical response at Day 3	41/125 (33)	34/125 (27)	0.334
Clinical response at EOAT	74/126 (59)	60/127 (47)	0.067
Clinical response at EOT	83/122 (68)	76/125 (61)	0.235
Outcome at short term FU visit (Day 28)			
New infection and/or relapse by Day 28*	8/44 (18)	5/31 (17)	0.865
Alive at Day 28	126/136 (93)	128/135 (95)	0.462

n–number of cases.

N–number of patients assessed for this outcome.

EOAT–end of allocated therapy; EOT–end of therapy; FU–follow up.

*- only patients with success at TOC were evaluated for new infection/relapses.

A total of 251 patients were evaluated at Day 28 either by on-site visit (66%) or by telephone interview (34%) (Fig 1). In the meropenem arm 9/61 (15%) and in the SOC arm 20/70 (29%) did not pass auditory tests (p = 0.057). No differences were observed in abnormal cerebral ultrasound—27/108 (25%) vs 30/110 (27%) in meropenem vs SOC arm, respectively.

General survival rate at Day 28 was 94% with no differences between study arms (Table 6). It was 98.8% (79/80) in Gram-positive and 90% (54/60) in Gram-negative infections. All but three patients who died had a BW <1200g.

### Rectal colonisation by CRGNO

The rectal swabs were available for 130, 101 and 95 patients in the meropenem and for 127, 94, 103 patients in SOC arm at baseline, EOT and Day 28/ NICU discharge visit, respectively. Cumulative acquisition of CRGNO by Day 28 was observed in 4/94 (4%) in the meropenem and in 12/101 (12%) in the SOC arm (p = 0.052) and highly CRGNO in 3/94 (3%) and 7/100 (7%), respectively. When comparing patients who had received at least one dose of meropenem (n = 170), regardless of study arm, with those not receiving meropenem, the acquisition of CRGNO in general or of highly resistant strains was similar—8/124 (6%) vs 8/71 (11%) for CRGNO and 5/124 (4%) vs 5/70 (7%) for highly CRGNO.

### Safety

A total of 193 patients (72%) had at least one AE. All cause AEs totalled 304 and 317, with 47 and 48 serious AEs in the meropenem and SOC arms, respectively. The AEs seen in ≥ 3% of patients are listed in Table 7. In the meropenem arm the most common AEs were anaemia, thrombocytopenia and meningitis and in the SOC arm anaemia, abdominal distension and apnoea. Seizures, a recognised side effect of carbapenems, were seen in four (3%) patients in the meropenem arm and one (<1%) in the SOC arm. Renal failure occurred in three (2%) patients in the meropenem arm and in four (3%) patients in the SOC arm.

**Table 7:** Comparative safety and presence of most common major clinical diagnoses in meropenem and SOC arm.

	Meropenem N = 136 (%)	SOC N = 132 (%)	P
Total number of patients with AE	91 (67)	102 (77)	0.059
Total number of patients with grade 3/4 AEs*	51 (38)	61 (46)	0.148
Total number of patients with SAEs	28 (21)	18 (14)	0.131
Discontinued treatment due to AEs	8 (6)	7 (5)	0.796
AE observed in more than 3% patients			
Anaemia	15 (11)	24 (18)	0.097
Thrombocytopenia	12 (9)	5 (4)	0.091
Meningitis	11 (8)	5 (4)	0.137
Abdominal distension	5 (4)	10 (8)	0.165
Oliguria	5 (4)	4 (3)	1.000
Apnoea	6 (4)	11 (8)	0.188
Respiratory distress	5 (4)	3 (2)	0.723
Sepsis	4 (3)	7 (5)	0.330
Oxygen saturation decreased	4 (3)	7 (5)	0.330
Seizures	4 (3)	1 (1)	0.622
Hyperglycaemia	3 (2)	7 (5)	0.212
Major clinical diagnoses in premature neonates			
RDS or HMD	53 (39)	62 (47)	0.186
PDA requiring surgery	37 (27)	37 (28)	0.880
Anaemia prematurity	33 (24)	37 (28)	0.483
Bronchopulmonary dysplasia	27 (20)	31 (23)	0.470
Apnoea of prematurity	24 (18)	35 (27)	0.080
Intracranial bleeding	21 (15)	24 (18)	0.548
NEC stage II or worse	11 (8)	16 (12)	0.273

*as defined in Common Terminology Criteria for Adverse Events v3.0 (CTCAE); published 9.August 2006 (http://ctep.cancer.gov)

## Discussion

We have performed a large RCT on the efficacy of antibiotics in LOS, undertaken in a population of predominantly premature, critically ill hospitalized neonates in Europe. We have shown that the mortality was low with both antibiotic regimens and the efficacy of meropenem was similar to commonly used SOC combinations based on a composite primary endpoint in the FAS population. If only patients with culture proven LOS were analysed the efficacy of meropenem was significantly greater than that of SOC in general but there were no differences between study arms if Gram-positive and Gram-negative sepsis were evaluated separately. Furthermore, patients randomised to meropenem had a shorter duration of antibacterial therapy than those randomised to SOC. The two study arms were similar in terms of adverse events and acquired perirectal colonisation by CRGNO.

The NeoMero1 study differed from previous studies in LOS in many ways. First, it was a multicentre study including countries with low to moderate antibiotic resistance rates (http://ecdc.europa.eu/en/healthtopics/antimicrobial_resistance/database/) in contrast to previous single center and/or national studies [5, 19]. Second, the demanding inclusion criteria resulted in recruitment of a very sick patient population (e.g. 55% mechanically ventilated, 35% with BW of <1000g) compared to previous studies [5]. Third, only 2% of patients were ineligible (did not have LOS) and altogether 52% had culture proven LOS as opposed to 15% in a recent study of complicated intra-abdominal infections [19]. Fourth, NeoMero1 had an ambitious primary endpoint that in addition to resolution or significant improvement of clinical and laboratory criteria, did not allow any changes of allocated therapy such as deviations from fixed treatment duration, dosing and/or addition of another antibiotic, in contrast to more liberal or less specific endpoints in previous studies [5, 19]. This ambitious primary endpoint most likely explains a low efficacy rate in terms of composite endpoint (23% in SOC vs. 32% in meropenem) in NeoMero1 study.

The most intriguing finding of this study, in comparison to others, was a relatively low success rate in terms of the composite primary endpoint in both study arms (23% in SOC vs. 32% in meropenem), The low efficacy rate was mainly driven by the modification of allocated therapy and most of all by its fixed duration of 8 to 14 days. The effect of the latter was clearly demonstrated in the post-hoc analysis in which changing the allowed treatment duration by just one day (from 8–14 days to 7–14 days) improved the success rate from 32% to 48% in the meropenem and from 23% to 27% in the SOC arms. We believe that this was due to the clinicians’ decision to stop antibiotics earlier than the pre-defined duration, presumably because they felt that clinically the sepsis episode had resolved and the infant had recovered. The optimal duration of antibiotic therapy in LOS is not known [23].

Despite low efficacy rate in terms of primary endpoint the mortality in NeoMero1 was low– 6% in total and 10% in Gram-negative sepsis. This is much lower much lower than in previous observational studies of LOS (15%-18% in total and 26%-36% in Gram negative sepsis [2, 3] and in a recent Egyptian study comparing conventional and prolonged infusion of meropenem [24] suggesting good efficacy of both antibiotic regimens.

In contrast to previous studies, we did not find an association between carbapenem use and CRGNO colonization [25–27]. Of note, our study was an RCT with strict inclusion criteria, in contrast to previous retrospective and/or observational studies, which included all patients without restriction [25, 27, 28]. We should emphasize that the relatively short duration (median of 9 days) of meropenem treatment in the NeoMero1 study may be relevant. For example, Clock *et al*. (2016) showed in an observational study that perirectal colonisation with Gram-negative multi-drug resistant bacteria was associated with >10 days of meropenem treatment [18].

In line with previous studies, meropenem was well tolerated and all AEs in this very sick patient population were well balanced between study arms [19]. Seizures, previously reported to be related to meropenem treatment [29] were seen in higher numbers in the meropenem arm but due to very low numbers no meaningful conclusions can be drawn.

The study had significant limitations. First, it was an open label study with the risk of investigator -induced bias when evaluating the primary endpoint or changing allocated therapy. An open label design was selected because meropenem monotherapy was to be compared with a combination of multiple comparator agents. Using a dummy infusion in critically ill, premature babies adds significantly to the complexity and cost of a multicenter trial and is questionable from an ethical perspective. We also note that the most appropriate targets for meropenem are Gram-negative microorganisms, including extended spectrum beta-lactamases (ESBL) or AmpC producing organisms. Despite the demanding inclusion criteria, that well discriminated between patients with and without LOS, these criteria performed poorly in distinguishing between cases caused by Gram-positive and Gram-negative microorganisms; about half of the recruited patients still had Gram-positive infections. As long as rapid and reliable methods or biomarkers, which allow differentiation between different species, are not available, recruitment of mixed population into similar empiric sepsis trials is unavoidable. To target antibiotic therapy more precisely, rapid and reliable tests that enable identification of microorganisms and/or their antibiotic resistance, and biomarkers that differentiate between infections and other illnesses, are urgently needed.

NeoMero1 is the largest European RCT for LOS since the 1970s [5, 6] but several outstanding issues require further studies to be done. For example, the question of best treatment options for LOS in developing countries and/or in areas with high antibiotic resistance rates was not addressed as 92% of microorganisms were susceptible to meropenem and 72% at least to one component of SOC. As shown by us, RCTs in LOS treatment are challenging due to a vulnerable population and lack of validated disease criteria and endpoints [5, 6, 30]. There is an urgent need for cooperation between academia, pharmaceutical industry and regulators in innovating clinical research in neonatology, including defining alternative and more feasible study designs (e.g. pharmacokinetics/pharmacodynamics, rather than solely clinical endpoint based designs, enabling modelling/simulation and extrapolation from studies in adults) [6, 30]. It is critical to provide efficacy data for those infected with organisms covered specifically or exclusively by study antibiotics (e.g. ESBL or AmpC producing organisms).

Only six patients did not have LOS suggesting that the LOS criteria developed by an European Medicines Agency expert group [5] were able to discriminate well between patients with and without LOS, but further improvement and validation of these criteria is needed before adopting and implementing them into clinical trials. Indeed, other definitions have been published, which use fewer clinical and laboratory parameters, but to the best of our knowledge, these have not been tested or used in large RCTs [30]. The recent STROBE-NI consensus for reporting neonatal sepsis trials should help with this in the future [31].

## Conclusion

In predominantly premature critically ill infants with LOS in Europe, we found no evidence that meropenem treatment was superior to SOC in terms of success at TOC. Short-term hearing disturbances, safety or mortality were similar in both treatment arms. However, meropenem monotherapy resulted in slightly shorter treatment duration and was superior to SOC in culture proven LOS. Meropenem did not lead to enhanced colonization with CRGNOs. We recommend that meropenem should be reserved for seriously ill premature neonates with suspected or proven Gram-negative LOS, especially in NICUs in which microorganisms producing ESBL and AmpC beta-lactamases are circulating.

## Supporting information

S1 Checklist CONSORT 2010 checklist of information to include when reporting a randomised trial*. (DOC)
